# Orchestrating an immune response against cancer with engineered immune cells expressing αβTCRs, CARs, and innate immune receptors: an immunological and regulatory challenge

**DOI:** 10.1007/s00262-015-1710-8

**Published:** 2015-05-20

**Authors:** Moniek A. de Witte, Guido J. J. Kierkels, Trudy Straetemans, Cedrik M. Britten, Jürgen Kuball

**Affiliations:** Department of Hematology, University Medical Center Utrecht, Room Number Q05.4.301, PO Box 85500, 3508 GA Utrecht, The Netherlands; Laboratory of Translational Immunology, University Medical Center Utrecht, Utrecht, The Netherlands; Translational Oncology, University Medical Center of the Johannes Gutenberg University Mainz (TRON), Mainz, Germany; Immuno-Oncology & Combinations DPU, Oncology R&D, GSK, Stevenage, UK

**Keywords:** Allogeneic stem cell transplantation, Innate immune system, Gene therapy, CIMT 2014, Phase I/II clinical trials, Regulatory authorities

## Abstract

Over half a century ago, the first allogeneic stem cell transplantation (allo-SCT) initiated cellular immunotherapy. For several decades, little progress was made, and toxicity of allo-SCT remained a major challenge. However, recent breakthroughs have opened new avenues to further develop this modality and to provide less toxic and equally efficient interventions for patients suffering from hematological or solid malignancies. Current novel cellular immune interventions include ex vivo expansion and adoptive transfer of tumor-infiltrating immune cells or administration of drugs which antagonize tolerizing mechanisms. Alternatively, transfer of immune cells engineered to express defined T cell receptors (TCRs) and chimeric antigen receptors (CARs) has shown its potential. A valuable addition to ‘engineered’ adaptive immunity has emerged recently through the improved understanding of how innate immune cells can attack cancer cells without substantial side effects. This has enabled the development of transplantation platforms with limited side effects allowing early immune interventions as well as the design of engineered immune cells expressing innate immune receptors. Here, we focus on innate immune interventions and their orchestration with TCR- and CAR-engineered immune cells. In addition, we discuss how the exploitation of the full potential of cellular immune interventions is influenced by regulatory frameworks. Finally, we highlight and discuss substantial differences in the current landscape of clinical trials in Europe as compared to the USA. The aim is to stimulate international efforts to support regulatory authorities and funding agencies, especially in Europe, to create an environment that will endorse the development of engineered immune cells for the benefit of patients.

**Electronic supplementary material:**

The online version of this article (doi:10.1007/s00262-015-1710-8) contains supplementary material, which is available to authorized users.

## Introduction

With the first allogeneic stem cell transplantation (allo-SCT) more than 50 years ago, a new era of therapeutic intervention was born, namely cellular immunotherapy. In particular, donor lymphocyte infusions (DLI) provided early and important insights into the potency and mode of action of immunotherapy, as it has the potential to induce sustainable remissions even in patients with advanced hematological malignancies. Also, solid malignancies can be targeted by T lymphocytes, both from an allogeneic stem cell source [1] and by endogenously derived tumor-infiltrating lymphocytes (TIL) [2]. Analyzing failure and success of immunotherapies in hematological malignancies, and in solid cancers, frequently elucidated the same requirements for an efficient therapy, i.e., the immune system seems to be most effective when mounting a complex immune response against a defined intruder. Yet, limiting immune interventions to one or two antigens in therapeutic interventions may increase the likelihood of tumor escape. This might explain the success of allo-SCT and TILs but also the recent clinical success of antibodies designed to act on inhibiting regulatory components of the immune system such as anti-PD1 or anti-CTLA4 [3], [4]. However, as ‘releasing the brake’ from all T cells does not only affect tumor-specific immune responses, unwanted ‘off-target’ reactivity is frequently observed, like graft-versus-host disease (GVHD) or immune-related side effects [4]. Consequently, ideal future designs of immune therapeutic interventions should broaden tumor antigen-specific immune responses, but without substantial toxicity.

A recent and promising intervention is the controlled enlargement of the immune repertoire by transferring tumor specificity. This transfer is accomplished by redirecting T cells with a receptor-recognizing defined antigens on a cancer cell [5]. Receptors explored to date have been either isolated from cancer reactive αβT cells [6] or engineered by fusing tumor-reactive antibodies with signaling domains of T cells, so-called chimeric antigen receptor (CAR) [7]. Landmark clinical trials with an TCR specific for MART-1 melanocyte differentiation antigen [8] or an anti-CD19 CAR [9] have shown the great potential of this approach, leading to an impressive number of ongoing clinical trials (Table 1 and Supplementary Table 1 insert ‘in ESM’). However, the number of antigens that can be safely targeted in patients is—at least at this stage—still relatively limited. In this view, the transfer of an alternative set of immune cells and receptors will be discussed. This includes the prospects of ‘low-GVHD’ transplantation protocols, based on the preservation of innate immune cells early after transplantation, which can serve as platform for additional immune interventions, as well as the transfer of immune cells designed to express highly selected (innate) immune receptors originated from the innate immune system such as NKG2D or γδTCRs (reviewed in detail [10]). These innate receptors are a less utilized type of immune receptors but possess some appealing and unique advantages as compared to TCRs and CARs. Finally, the prospects and limitations of broadening the application of this exciting and potent therapeutic strategy are discussed.

**Table 1:** Ongoing clinical trials with TCR- or CAR-modified T cells

	Targeted antigens	Stem cell source	Number of trials in USA	Number of trials in EU
TCR based (*n*=13)	NY-ESO-1 (*n*=6); MAGE-A3 (*n*=2); WT-1 (*n*=2); MART-1 (*n*=1); miscellaneous (*n*=2)	Autologus (12); unknown (1)	12	1
CAR based (*n*=52)	CD19 (n=27); GD2 (*n*=4); mesothelin HER2 (*n*=3); miscellaneous (*n*=14)	Autologus (49); allogenic (4)	47	5

## Innate allo-SCT as a novel immune platform for early immune interventions

**Fig. 1:**
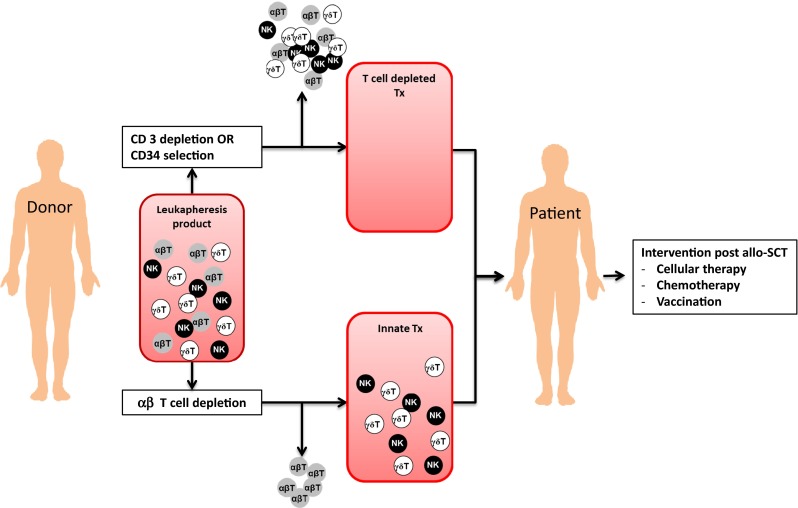
Low GVHD allo-SCT platforms can be achieved by complete removal of the T cell repertoire via CD34+ selection [13] (*upper part*). Alternatively, a so-called innate allo-SCT can be generated by specific removal of T cells from the graft, rendering γδT cells and NK cells within the leukapheresis product (*lower part*) [14], [15]

Allo-SCT substantially increases the overall survival for many patients with high-risk hematological diseases. Nevertheless, the outcome for most patients is still poor, due to the high risk of developing severe life-threatening GVHD, encountering relapse, or substantial long-term toxicity with a reduced quality of life. Adoptive transfer of genetically modified T cells with a tumor-specific TCR is therefore an attractive strategy to skew the T cell compartment toward a more defined anti-tumor repertoire post-allo-SCT [11]. As such, there is a need for less toxic transplantation regimens which have a substantially reduced incidence of GVHD, do not require long-term immune suppression, and allow for early additional immune interventions. This may be achieved by separating the initial engraftment of stem cells from the application of immune cells via partial or complete removal of immune cells from the allograft prior to transplantation (Fig. 1). These transplantation strategies, with either a delayed endogenous T cell reconstitution or a T cell add back via DLI, have resulted in a decreased transplantation-related mortality (TRM) due to a lower incidence of GVHD, as compared to T cell replete allo-SCT [12]. This is a consequence of separation of the inflammation mediated by the required conditioning from the infusion of αβT cells. For instance, in a recent prospective and randomized phase III clinical trial, Pasquine et al. [13] have demonstrated that complete elimination of immune cells by enrichment of the CD34^+^ cells lowers long-term morbidity as a result of a substantially reduced chronic GVHD, without negatively impacting relapse rates in patients with acute myeloid leukemia (AML). Selective depletion of αβT cells has been suggested as an alternative approach [14]. This strategy maintains NK cells and γδ T cells in the graft, which have an intrinsic activity against tumors and infections, without detrimental reactivity toward healthy tissue [10]. As this regimen favors the early reconstitution of the innate immune system, such a strategy should theoretically result in an improved control of the tumor and infections. The feasibility of such an approach has been shown most recently by Bettiana et al. [15], in which 23 children with non-malignant disorders received a HLA-haploidentical hematopoietic stem cell transplantation (haplo-HSCT) after ex vivo elimination of T cells and CD19^+^ B cells. In this cohort, none of the patients developed a GVHD ≥grade III, and the cumulative incidence of TRM was 9.3 %. However, the impact on malignancies could not be assessed, as only those patients with benign disorders received transplantations. Currently, T cell depletion (Fig. 1) is evaluated both in the setting of haplo-SCT and in the MRD/MUD in patients suffering from hematological malignancies. A potential drawback of these strategies is that innate immune system reconstitution early after transplantation is very diverse and does not necessarily contain all the components required to control tumors and infections (for review see [16]). In addition, even with an optimally selected donor, the innate immune system quickly becomes accustomed to its new host environment (education), resulting most likely in a loss of efficacy of the innate donor immune system after a couple of months post-allo-SCT [17]. This creates the need for additional immune interventions that do not increase GVHD after an innate allo-SCT, in particular within the context of poor risk hematological malignancies, for which the race against relapse is difficult to win, and GVHD remains a substantial threat.

## Moving from DLI to genetically engineered T cells: aiming for a diverse repertoire with multiple and complementary defined receptors

To date, the most potent immune intervention after allo-SCT is a DLI. DLI has already for some decades been appreciated as a curative treatment in relapsing disease after allo-SCT, especially in patients with chronic myeloid leukemia, and to a lesser extend for AML [18], [19]. Also for hematological diseases, originally thought to be less sensitive to DLI—like acute lymphoblastic leukemia (ALL) and multiple myeloma (MM)—some recent reports have shown a beneficial effect of DLI [20], [21]. Effects are usually observed 4–6 weeks post-application, after doses administered with a range between 1 × 10^5^ and 1 × 10^7^ T cells per kg. However, a DLI does not always provide tumor control, and DLI can be associated with substantial GVHD. Again the unpredictable diversity in the repertoire of a DLI is the major hurdle, given that the dose of the tumor-reactive T cells within the DLI is most likely just a fraction of the total T cell pool, as the frequency of allo-reactive T cells is reported to be between 1–10 % [16].

**Fig. 2:**
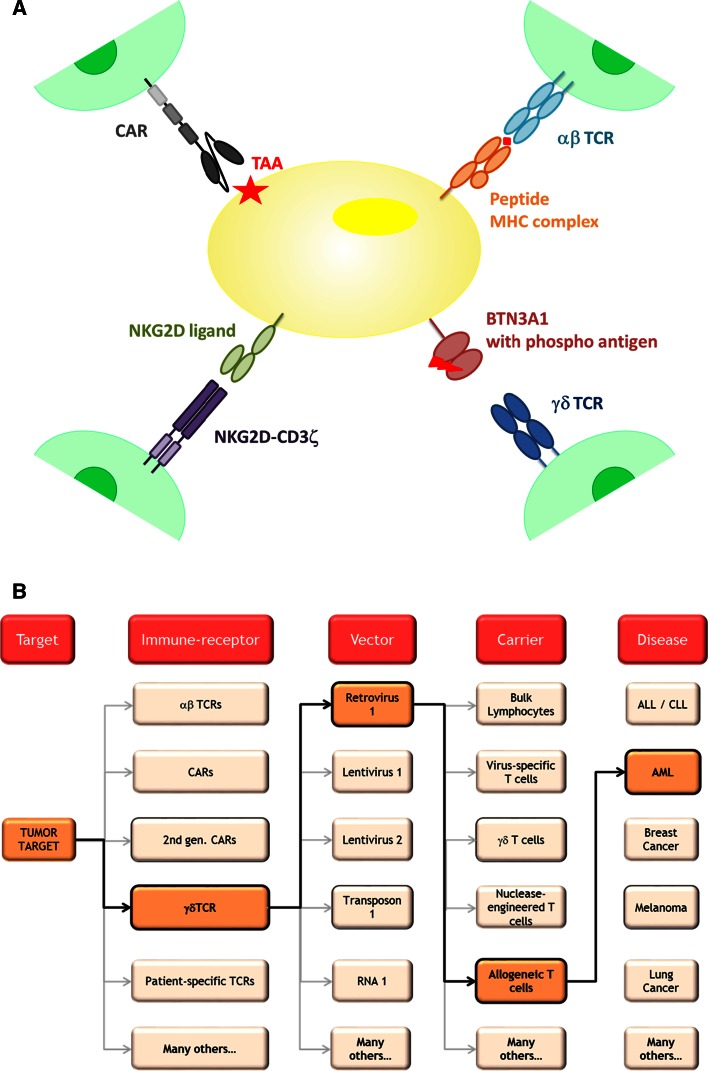
**a** Utilization of T cell receptors, T cell receptors, CARs, and NK cell receptors to transfer desired immune specificities to donor T cells. *TAA* tumor associated antigen. **b** Toolbox of immune receptors, vectors for gene transfer, and carrier cells that can be combined with each other to treat different malignancies

In order to further increase efficacy of a DLI while reducing toxicity, limitation of the diversity of transferred cells is needed. Most likely, relatively low doses will be sufficient, given they have the correct specificity and are available within a defined immunological subtype which can expand, contract, and provide long-term memory. In addition, some variety must be preserved to allow a diverse repertoire to tackle cancer cells at different targets and to prevent tumor escape mechanisms, such as antigen loss. This goal can potentially be accomplished by taking advantage of T cells genetically engineered to express a single or a variety of diverse tumor-specific immune receptors (Fig. 2a). Already over a decade ago, it has been demonstrated in animal models that the specificity of a T cell can be transferred between T cells by introduction of and TCR genes [22]. These and other observations have been translated to the first series of clinical trials with TCR-transduced T cells with different target antigens for solid malignancies [23]. Tumor-reactive TCRs were classically either isolated from TILs, from peripheral blood of patients responding to immune therapy (MAGE vaccination studies for instance), or from mouse origin. As a consequence, isolated anti-tumor TCRs are restricted toward a limited pool of HLA molecules. To further extend this method to have broader application, it may be technically feasible to generate cellular products harboring multiple tumor-specific immune receptors extracted from a given patient. With innovative techniques, in which the cancer exome is analyzed in a high throughput fashion [24], it is now possible to identify tumor-specific T cells directed against unique tumor antigens in individual patients [25] and as such fully exploit the cancer ‘antigenome’ and overcome HLA barriers. In addition to neo-antigen TCR transfer, novel treatment concepts may arise from identifying highly abundant TCR pairs from TILs, as they seem to be enriched for tumor mutation-specific antigens (unpublished data presented by S. A. Rosenberg at AACR 2014 in San Diego). Although transfer of neo-antigen-specific TCRs and TCR gene capture may bear a huge potential, such a personalized treatment concept will face major medical, regulatory, logistical, and financial challenges, as it creates the need to individualize genetic engineering to multiple (known and unknown) targets varying for every given patient.

CARs—which can be applied irrespective of HLA type—seem a highly attractive alternative for clinical implementation by pharmaceutical companies. The first clinical studies with a CD19-specific CAR have shown very promising results in ALL [26] and CLL [9] and led to an impressive amount of clinical trials (Table 1 and reviewed in [27]). The results of these studies will provide valuable information which is likely to contribute to the improvement in cellular therapy. However, the number of antigens for which antigen-specific receptors are tested in current clinical trials is frequently redundant and thus very limited (as shown in Table 1). To expand cellular therapy to a broader range of tumors or to enlarge the TCR- or CAR-redirected T cell repertoire, alternative targets and receptors are needed. Receptors of the innate immune system might provide an interesting alternative (Fig. 2) [10], [16].

## Innate immune receptors with unique features leading to comprehensive tumor recognition

Natural killer (NK) cells are the most widely studied subset of innate immune cells in the context of anti-tumor responses. NK cells express an array of activating and inhibitory receptors, which collectively discriminate healthy cells from diseased cells by sensing self-‘stress’ molecules on diseased target cells, including tumor cells [28]. NKG2D is the best known of these receptors. NKG2D recognizes stress-induced self-MHC class I-related proteins, which have a selectively increased surface expression on transformed cells from both hematological and solid origins [29]. To harness NK cell-mediated toxicity, chimeric receptors linking NKG2D to the cytoplasmic domain of CD3ζ have been constructed, and T cells equipped with such an NKG2D receptor display anti-tumor reactivity in both hematological and solid tumor models [30]. Also, bi-specific antibodies of a NK cell receptor fused to a single-chain fragment have shown tumor reactivity in various tumor models [31]. Inhibitory receptors impede NK cell reactivity by sensing the presence of MHC class I molecules constitutively expressed on almost all healthy cells. Killer cell immunoglobulin-like receptors (KIRs) are a well-studied example of such inhibitory molecules. For example, it has been reported that NK cells can kill allogeneic cells when their inhibitory KIRs are not engaged due to mismatched HLA alleles [32]. Two recent phase I studies in AML [33] and MM [34] have shown that an anti-KIR antibody can be safely administered to patients, and as such, full KIR saturation can be achieved, supporting subsequent trials to test for clinical activity.

Following NK receptors, the γδTCR has recently drawn attention as an alternative anti-tumor immune receptor with some unique appealing features (reviewed in [10]). γδ T cells express a somatically recombined γδ TCR, but behave like innate cells in a way that they—like NK cells –become activated by ‘stressed cells,’ The γδTCR is just one of the multiple proteins on the surface of a γδ T cell which can sense molecular stress signatures. A significant subset of γδ T cells express a TCR composed of V9 and V2 chains, which can recognize multiple targets on malignant cells, such as the complex of apolipoprotein A1 (ApoA1) and F1-ATPase. In addition, they can sense accumulated non-peptidic pyrophosphate molecules (phosphoantigens), intermediates of a deregulated mevalonate pathway of isoprenoid synthesis, via BTN3A1 (CD277). As such, γ9δ2T cells can mount immune responses against tumor cells derived from both hematological and solid malignancies [35], [36]. Unfortunately, translating these in vitro observations into effective clinical protocols remains challenging, since—despite substantial evidence in vivo in mice [37]—adoptively transferred autologous γδ T cells demonstrate anti-tumor reactivity only at modest and variable response rates [16]. The moderate success of these responses seems to be critically determined by the composition of the γδ T cell repertoire. Diversity in the γδTCR as well as the co-receptor repertoire leads to a diverse function and activation status of an individual γδ T cell. This makes ‘the γδ T cell repertoire’ a very heterogeneous population with anti-tumor activity that is difficult to predict. For instance, in vitro analysis of individual γ9δ2T cell clones revealed a highly differential anti-tumor reactivity [38], which did not appear to be explained by the different repertoire of inhibitory and activating receptors on individual cells [39], but by the small variations in the CDR3 region of the γ9δ2 TCR [38].

Therapeutic concepts with engineered ‘innate receptors’ fall in two categories: single proteins and membrane-bound receptors, and both may complement other types of cellular therapies. For instance, NKG2D fused to anti-CD3 variable fragment (scFv-NKG2D) has been shown to engage tumor cells with T cells [34]. Also antibodies directed against KIRs (see above) and soluble MHC class I-related protein A (sMICA; a ligand for NKG2D which in its soluble form is associated with NK inactivation) have been demonstrated to stimulate T cell-specific responses [40]. Concomitant administration of such proteins, with DLI or an engineered cellular product, may very well result in synergistic responses. The group of membrane-bound engineered innate receptors can either consist of (optimized) wild-type protein [38], fusion proteins (CARs) or single-chain receptors. Transfer of such receptors into T cells may complement attractive features of both the innate and adaptive immune system. T cells are easy to collect and are fully equipped to proliferate and activate upon antigen recognition. The introduced innate receptor is not MCH-restricted. In addition, the formation of mixed dimers does not occur. As such, a cellular product can be engineered containing sufficient numbers of effector cells with desired and uniform specificity.

For example, our group is preparing a phase I trial for γδTCR gene transfer. To ‘pick the most effective γδTCRs’ for future clinical applications, we have developed the ‘combinatorial γδTCR chain exchange.’ This allows for selection of the γ9δ2TCRs with the highest affinity [38]. Like their counterparts, γδTCR genes can be retrovirally transduced into both CD4^+^ and CD8^+^ T cells. These γδTCR-engineered T cells can recognize a broad panel of tumor cell lines both in vitro and in vivo as well as a variety of primary AML blasts, but they ignore non-transformed cells [41]. Interestingly, introduction of the γδTCR leads to down-regulation of the endogenous TCR, likely due to competition for components of the CD3 complex. This competition can be used to negatively select non-engineered cells with TCR ^bright^ cells from a transduced T cell bulk, resulting in an end product containing almost 100 % γδTCR-engineered T cells with increased anti-tumor function in vitro as well as in vivo (Straetemans et al, unpublished data). These results have led to the design of the first clinical trial in which γδ TCR-engineered T cells will be tested in patients with AML, who will receive an T cell-depleted stem cell transplantation from a MRD/MUD (Fig. 1) followed by an infusion of γδ TCR-engineered T cells 3–6 months post-allo-SCT.

## A space of endless choices: How to develop the best cellular immunotherapy?

Further work is needed to establish genetically modified T cells as a widely accepted mode of treatment for hematological malignancies. Preclinical and small phase I studies—in the past mainly initiated by academic institutions, but now increasingly promoted by young and innovative biotech companies—are essential to broaden existing concepts and to develop new concepts. While the field is moving from allo-SCT to more engineered cellular products that are enriched for anti-tumor activity and depleted of unwanted cross-reactive T cells, many challenges remain for the translation of novel concepts from the lab to the clinical setting.

Engineered cellular therapies constitute a new class of products that on one hand bear a huge potential for benefit (as shown by unprecedented effect sizes in a large fraction of treated patients) but on the other hand bear a risk of serious (even fatal) side effects [42]. As outlined above, earlier progress in science and technologies has equipped us with a huge toolbox of immune receptors, vectors for gene transfer, and carrier cells that can now be combined with each other (or with additional non-cellular compounds) in multiple ways and therefore give rise to countless different permutations of products for the treatment of various hematological and solid cancers (Fig. 2b).

Altering one component of the toolbox could lead to increased benefit or increased toxicity (or both) for patients. Due to the species specificity of antigen expression, antigen processing, antigen presentation, and immune recognition, the available non-clinical in vivo models are not predictive for the outcome in patients [43]. Although non-clinical in vitro studies to predict off-target cross-reactivity have been proposed [44], it is clear that the final answers can only be obtained in clinical trials. A researcher may feel inclined to utilize an empiric approach and iteratively test multiple permutations in a series of small-scale trials, to identify the more toxic products as early failures and to select the best therapies for advanced clinical testing. Given that each permutation of the tool box is considered as a novel compound, and due to the high regulatory requirements associated with clinical testing of each permutated product, the described “empiric approach” will simply not be feasible. Due to limitations in time and available resources, this approach will only allow testing of one (or very few) permutations in the clinic.

In this light, the existing regulatory framework requires a lively discussion of the field on what might be done to facilitate the access to innovative cellular therapies without increasing the risk for patients. Some investigators have raised the criticism that the field of cellular immunotherapy (in particular therapies that include engineering of lymphocytes with TCRs or CARs) is sometimes held to higher standards as compared to other and more established groups of products. One seemingly well documented example is the requirement to test for replication competent retrovirus (RCR) which sponsors have to do on the master cell banks, retroviral supernatant lots as well as on the actual T cell product [45]. FDA guidelines further require follow-up analysis for RCRs to be performed at 3, 6, and 12 months and yearly following treatment. The required RCR testing is labor-intensive, costly, and time consuming, which limits the translation of such innovative approaches. Currently, available data from more than 500 patient-years of clinical implementation did not show any evidence for secondary malignancies due to insertional mutagenesis form retroviral gene transfer to lymphocytes in patients [46]. It seems difficult to understand why developers of gene/cell therapies have to meet this high level of testing requirements despite the availability of decade-long safety experience, while chemotherapeutic agents that have a documented rate of induction of secondary malignancies may be used in thousands of patients every day without similar testing requirements. In addition, there is great uncertainty how to best design non-clinical programs to support clinical trial applications. Given the potentially low predictive value of animal models and the availability of an increasing repertoire of in vitro tests to predict potential toxicity in humans (e.g., alanine scans for novel TCRs or use of complex tissue cell cultures), now may be the time to delineate minimum requirements for non-clinical programs that both developers and regulators may agree upon. Such efforts cannot be achieved by single investigators but require field-wide efforts and a consensus process that can only happen in larger networks with broad representation of the different stakeholders.

Challenges are even more complex when considering the novel personalized treatment concepts with patient-specific neo-antigen-specific TCRs and TCR genes captured from TILs. These strategies can be regarded as personalized therapies (similar to mutanome vaccines) where the specificity of the drug product will change between patients [47]. If the same regulatory requirements for these personalized products are applied as for defined products, these new treatment concepts will never become feasible. Thus, a novel regulatory blueprint is needed for such personalized TCR approaches. A first justification for a less stringent handling of such personalized products could arise from the position that (similar to a DLI) no new specificity is added to the immune system. Still, a series of unresolved regulatory questions exists and needs to be solved prior to testing these novel therapies in the clinic. As mentioned earlier, the delineation of applicable principles for such a novel and disruptive type of personalized medicine needs to be accomplished through scientific discussions among the various stakeholders and incorporates a balance between the patient’s interest for safe but also timely access to novel treatments. A similar strategy has also been proposed to define a first regulatory blueprint for mutanome vaccines [47].

In summary, there is an urgent need for innovative strategies to reduce the amount of time and resources for bringing a novel cell therapy into the clinic without inappropriately increasing the risk for patients. Such strategies would allow for testing more combinations of novel technologies and realization of their full potential. We propose to enter an open discussion about the generation of flexible regulatory frameworks tailored to the unique properties of immune receptor-engineered cells. The major challenge will be to balance the need for safety within the context of urgent medical need, which requires continuous innovation.

## A tenfold difference in clinical trials with genetically engineered T cells, what is causing the gap?

To determine which types of products are currently developed, a literature search was performed for ongoing clinical trials with TCR- or CAR-modified T cells. We identified an encouraging number of 65 studies, of which only a disappointing 10 % were enlisted in the European Union (EU) (Table 1). The remaining 90 % were listed in the USA.

One may only speculate on the reasons for this striking difference. Critical success factors that are often discussed but that never seem to be systematically addressed are large clinical/academic infrastructures, access to funding for innovation not only in the early stage but also for clinical trials, and concentration of talent which typically moves to the most attractive environments. Regarding the first, a variety of lists are published yearly, ranking the top universities around the world. The bulk of the top 10 is situated in the USA. Many of the currently developed cellular immunotherapies emerged from large institutions such as the NIH, the University of Pennsylvania, or the MD Anderson Cancer center that combine access to patients, scientific infrastructure, medical expertise, manufacturing capacities, and funding opportunities in a way that is difficult to match at most European institutions which are much smaller in size. Comparing (financial) resources between the USA and Europe seems virtually impossible as well. Within Europe, many national and international governmental, private, and commercially sponsored programs exist. As an example, since 1984, the European Commission has launched seven framework programs, which are dedicated to research and innovation. Since 1998, 351.1 million EUR has been donated to programs involving gene therapy projects [48]. This year, the eighth framework program Horizon 2020 opened, which is the biggest EU research and innovation program ever, with nearly 80 billion of funding available over 7 years up to 2020. It aims to secure Europe’s global competiveness by ensuring that Europe can produce world-class science, remove barriers to innovation, and make it easier for the public and private sectors to work together in delivering innovation. However, as impressive as the numbers might be at a first sight, initiatives like these seem very modest when compared to resources available in the USA, where young and innovative companies such as Juno or Kite can raise several hundred million dollars within a very short time. Another example is the number of family foundations in the USA that has grown from about 3200 in 2001 to more than 40,000 in 2015, with total annual grants for academic research and translation of more than $21.3 billion, according to the Foundation Center. In the USA, federal tax breaks encourage the funding through foundations, which give philanthropists more control over their donations. A third—and perhaps more fundamental—hurdle in initiating clinical trials in Europe with genetically modified cells is the bureaucratic burden imposed by regulation. In 2004, the European Clinical Trials Directive 2001/20/EC (EU-CTD) was introduced in order to protect clinical trial subjects by establishing quality, safety, and ethical criteria of initiated trials. In current practice, this implies that a trial needs to be reviewed in each individual member state by both a research ethic committee and national competent authority. This process turned out to be suboptimal in daily practice. First, one could argue that a scientific and ethical judgment should be integrated in one review body, since the scientific merit of clinical research cannot be judged without an ethical evaluation and vice versa. In addition, leaving the organization of the review bodies to the individual member states leads to large inter-country differences and subsequent inconsistent evaluations (reviewed in [49]). Consequently, between 2007 and 2011, the number of clinical trials conducted in the EU fell by 25 %, and the number of clinical trials applied for in 2007 (5000) dropped to 3800 by 2011, with most studies being limited to one country. So despite a potential additional protection of subjects, the European Clinical Trial Directive seems to prohibit European citizens from accessing innovative therapies. In the USA, there is a more streamlined approach, with one nationally appointed service, namely the Department of Health and Human Services (DHHS) providing oversight of clinical trials. All investigators must comply with these regulations when conducting clinical gene therapy trials. Although precise numbers are absent, the general feeling among researchers in the field is that the time to approve a gene therapy protocol is shorter in the USA than it is in the EU. This may also be reflected by the already mentioned difference in numbers of clinical trials in the EU as compared to the USA. Also, a trend toward increased clinical trials in areas with emerging economies such as Asia, South America, and Russia is acknowledged by the EU.

To diminish the time-consuming bureaucracy in the EU, a new legislation ‘Clinical Trial Regulation EU No 536/2014’ was adopted on April 2014 and is scheduled to be implemented in May 2016. The objective is ‘to restore European Union’s competitiveness in clinical research and the development of new and innovative treatments and medicines for the ultimate benefit of patients.’ Indeed, they claim to make the process more transparent and faster, but the notion that a single research protocol still has to be judged both by a scientific committee of a reporting member state and separately by a scientific committee of each individual member state makes one wonder whether this new legislation will truly lead to a reduction ‘in red tape’ and increase in trials. Obviously, the efficacy of this new legislation has to be evaluated—which will at least take another year—, but the lack of centralization of the process feels like a missed opportunity, not only with regard to the speed of the process, but also as an incentive for European researches to act as a united team.

Regardless of the reasons for the striking differences in the processes for clinical trials initiated in the USA versus Europe, it is clear that researchers who intend to translate their science into novel therapies for patients should still mainly focus on the science around engineered cellular products. Additionally, researchers should also contribute to improving the regulatory, structural and financial cornerstones for these novel treatments.

## Concluding remarks

Designing novel concepts of allo-SCT by promoting the early reconstitution of innate immune cells complemented subsequently with ‘genetically engineered immunity’ by utilizing both, innate and adaptive, receptors has the potential to substantially reduce toxicity and provide a profound short- and long-term protection against cancer. Components of this concept can be utilized not only for hematological but also for solid malignancies and allow engineering of a diverse immune response against cancer. Bearing in mind that the associated treatment-related morbidity and mortality of the current state-of-the-art treatment for many poor risk hematological malignancies are substantial, regulatory, and financial requirements, for the implementation of novel innovative cellular designer drugs seem to be completely out of balance. With a treatment-related mortality of up to 30 %, frequently accepted for many routinely performed allo-SCTs in 2015 worldwide, one might question whether current regulatory and financial hurdles compromise rather than protect the lives of our patients. Thus, controlling cancer needs not only an orchestrated action of immune cells expressing defined CARs, αβTCRs, γδTCRs, and other innate immune receptors, but also a well-balanced discussion about regulatory and financial needs with all involved groups: the academic research community, pharmaceutical companies, authorities, and patients and their families—a major immunological and societal challenge.

## Electronic supplementary material

Supplementary material 1 (PDF 448 kb)

## Abbreviations

ALL: Acute lymphoblastic leukemia
Allo-SCT: Allogeneic stem cell transplantation
AML: Acute myeloid leukemia
CAR: Chimeric antigen receptor
CLL: Chronic lymphocytic leukemia
CML: Chronic myeloid leukemia
DHHS: Department of Health and Human Services
DLI: Donor lymphocyte infusion
EU: European Union
EU-CTD: European Clinical Trials Directive 2001/20/EC
GVHD: Graft-versus-host disease
haplo-HSCT: Haploidentical hematopoietic stem cell transplantation
KIR: Killer cell immunoglobulin-like receptor
MM: Multiple myeloma
MRD: Matched related donor
MUD: Matched unrelated donor
NK cells: Natural killer cells
NCR: NK cell receptor
RCR: Replication competent retrovirus
REC: Research ethic committee
SCT: Stem cell transplantation
TCR: T cell receptor
TIL: Tumor-infiltrating lymphocyte
TRM: Transplantation-related mortality
UMC: University Medical Center
