# Supplementary material for: Orchestrating an immune response against cancer with engineered immune cells expressing αβTCRs, CARs, and innate immune receptors: an immunological and regulatory challenge
Source: Cancer Immunol Immunother. 2015 May 20;64(7):893–902. doi: 10.1007/s00262-015-1710-8 (PMC4481298; doi:10.1007/s00262-015-1710-8)
Supplement: Supplementary file 1 — Supplementary material 1 (PDF 448 kb) [file 262_2015_1710_MOESM1_ESM.pdf]

Supplementary table 1, *de Witte et al*

Current and planned clinical trials with either $\alpha\beta$ TCR or CAR based gene therapy studies, both in an autologous as well as in an allogeneic setting within the USA and EU. Search method: Open studies in USA and EU, excluding studies with unknown status. Queries: TCR, CAR, chimeric antigen receptor. Excluding non-oncology conditions and excluding non-TCR/CAR methods. Search date: December 1st 2014

Sponsor	Country	ID	Phase	Type	Target	Method	Engineering	Start date	Completion date	Condition	Enrollment
Jonsson Comprehensive Cancer Center	USA	NCT00910650	II	TCR	MART-1	Autologous PBMCs<br>+ MART-1.26-35 peptide-pulsed dendritic cell (DC) vaccines	Retroviral vector	OCT09	DEC16	Metastatic Melanoma	22
Jonsson Comprehensive Cancer Center	USA	NCT02070406	I	TCR	NY-ESO-1	Autologous T-cells<br>+ ipilimumab<br>+ NY-ESO-1157-165 peptide pulsed DC vaccine	Retroviral vector	MAR14	FEB19	Unspecified Adult Solid Tumor	12
Jonsson Comprehensive Cancer Center	USA	NCT01697527	II	TCR	NY-ESO-1	Autologous PBMCs<br>+ NY-ESO-1157-165 peptide pulsed DC vaccine	Retroviral vector	NOV12	OCT18	Malignant Neoplasm	22
NCI	USA	NCT02111850	I/II	TCR	MAGE-A3	Autologous $CD4^+$ T-cells	Retroviral vector	JAN14	JAN17	Metastatic Cancer	107
NCI	USA	NCT02153905	I/II	TCR	MAGE-A3	Autologous T-cells	Retroviral vector	MAY14	NOV18	Metastatic Cancer<br>Metastatic Melanoma	102
NCI	USA	NCT02062359	II	TCR	NY-ESO-1	CD62L+ lymphocytes	Retroviral vector	FEB14	JUN19	Metastatic Cancer<br>Metastatic Melanoma	22
Abramson Cancer Center of the University of Pennsylvania	USA	NCT02030847	II	CAR	CD19	Autologous T-cells	Lentiviral vector	JAN14	JUL15	B Cell ALL	24
Adaptimmune	USA	NCT01350401	I/II	TCR	NY-ESO-1/LAGE	Autologous T-cells	Lentiviral vector	MAY11	FEB15	Melanoma	6

Supplementary table 1, *de Witte et al*

Sponsor	Country	ID	Phase	Type	Target	Method	Engineering	Start date	Completion date	Condition	Enrollment
Adaptimmune	USA	NCT01892293	I/II	TCR	NY-ESO-1c259	Autologous T-cells	Unknown	SEP13	APR15	MM	10
NCI	USA	NCT01967823	II	mTCR	NY-ESO-1	Autologous PBMCs	Retroviral vector	SEP13	MAR15	Metastatic Cancers Other Than Melanoma	43
Adaptimmune	USA	NCT01352286	I/II	TCR	MAGE-A3/6 NY-ESO-1	Autologous T-cells Syngeneic T-cells	Unknown	APR11	APR14	MM	26
Loyola University	USA	NCT01586403	I	TCR	TIL 1383I	Autologous T-cells	Unknown	JUL12	SEP13	Melanoma	15
Abramson Cancer Center of the University of Pennsylvania	USA	NCT02135406	I	CAR	CD19 scFv TCR ζ	Autologous T-cells	Lentiviral vector	MAY14	MAY16	MM	15
Fred Hutchinson Cancer Research Center	USA	NCT01640301	I/II	TCR	WT1	Unknown	Unknown	DEC12	MAR17	myelodysplastic syndrome AML, CML	55
Abramson Cancer Center of the University of Pennsylvania	USA	NCT01747486	II	CAR	CD19	Autologous T-cells	Unknown	DEC12	DEC15	chronic lymphocytic leukemia small lymphocytic leukemia	32
Memorial Sloan-Kettering Cancer Center	USA	NCT01430390	I	CAR	CD19	Allogeneic Epstein-Barr Virus Specific Cytotoxic T-Lymphocytes	Unknown	SEP11	SEP15	ALL	26
Abramson Cancer Center of the University of Pennsylvania	USA	NCT02030834	II	CAR	CD19	Autologous T-cells	Unknown	JAN14	JUL15	Non-Hodgkin Lymphoma Patients with CD19+B Cell Lymphomas	55
Abramson Cancer Center of the University of Pennsylvania	USA	NCT01355965	I	CIR (CAR)	mesothelin	Autologous T-cells	Unknown	MAY11	MAY14	Malignant Pleural Mesothelioma	6

Supplementary table 1, *de Witte et al*

Sponsor	Country	ID	Phase	Type	Target	Method	Engineering	Start date	Completion date	Condition	Enrollment
Baylor College of Medicine	USA	NCT01822652	I	CAR	GD2	Autologous T-cells	Retroviral vector	AUG13	AUG15	Neuroblastoma	14
NCI	USA	NCT02107963	I	CAR	GD2	Autologous T-cells	Retroviral vector	FEB14	DEC16	Sarcoma, Osteosarcoma, Rhabdomyosarcoma, Ewing Sarcoma, Melanoma	72
Abramson Cancer Center of the University of Pennsylvania	USA	NCT02209376	I	CAR	EGFRvIII	Autologous T-cells	Lentiviral vector	JUL14	JUL16	Glioma	12
NCI	USA	NCT01087294	I	CAR	CD19	Allogeneic T-cells	Retroviral vector	FEB10	OCT15	B-cell Leukemia<br>Hodgkin Lymphoma<br>Non-Hodgkin Lymphoma<br>B-Cell Lymphoma	36
Seattle Children's Hospital	USA	NCT01683279	I	CAR	CD19	Autologous T-cells	Lentiviral vector	DEC12	JAN15	B Cell Leukemia	18
Baylor College of Medicine	USA	NCT00902044	I	CAR	HER2	Autologous T-cells	Unknown	JUL09	JUL15	Sarcoma	36
Celdara Medical, LLC	USA	NCT02203825	I	CAR	NKG2D-Ligands	Autologous T-cells	Unknown	OCT14	OCT15	AML, MM<br>Advanced Myelodysplastic Syndrome	21
Memorial Sloan-Kettering Cancer Center	USA	NCT01840566	I	CAR	CD19	Autologous T-cells	Unknown	APR13	APR15	Non-Hodgkin Lymphoma	18
Baylor College of Medicine	USA	NCT02050347	I	CAR	CD19	Autologous T-cells<br>Allogeneic T-cells	Retroviral vector	APR14	MAR18	Non-Hodgkin's Lymphoma<br>B-Cell ALL<br>B-Cell CLL	56
Baylor College of Medicine	USA	NCT01953900	I	CAR	GD2	Autologous VZV T-cells	Retroviral vector	APR14	APR18	Sarcoma	26
Seattle Children's Hospital	USA	NCT02028455	I/II	CAR	CD19	Autologous T-cells	Lentiviral vector	JAN14	JAN17	CD19+ Leukemia	80
Baylor College of Medicine	USA	NCT01109095	I	CAR	HER2	Autologous CMV T-cells	Unknown	JAN14	JAN17	Glioblastoma Multiforme	18

Supplementary table 1, *de Witte et al*

Sponsor	Country	ID	Phase	Type	Target	Method	Engineering	Start date	Completion date	Condition	Enrollment
Baylor College of Medicine	USA	NCT00881920	I	CAR	kappa immuno-globulin	Autologous T-cells	Retroviral vector	JUL09	JUL19	Lymphoma<br>Myeloma<br>Leukemia	54
Baylor College of Medicine	USA	NCT01316146	I	CAR	CD30	Autologous T-cells	Unknown	DEC11	DEC14	Non-Hodgkin's Lymphoma<br>Hodgkin's Lymphoma	18
NCI	USA	NCT00924326	I	CAR	CD19	Autologous T-cells	Retroviral vector	FEB09	JAN17	Small Lymphocytic Lymphoma<br>Mantle Cell Lymphoma<br>Follicular Lymphoma<br>Large Cell Lymphoma<br>CLL	40
Abramson Cancer Center of the University of Pennsylvania	USA	NCT02159716	I	CAR	mesothelin	Autologous T-cells	Lentiviral vector	JUN14	JUN16	Metastatic Pancreatic (Ductal) Adenocarcinoma<br>Epithelial Ovarian Cancer<br>Malignant Epithelial Pleural Mesothelioma	24
NCI	USA	NCT01454596	I/II	CAR	EGFRvIII	Autologous T-cells	Retroviral vector	SEP11	SEP19	Malignant Glioma<br>Glioblastoma<br>Brain Cancer	160
Fred Hutchinson Cancer Research Center	USA	NCT01865617	I/II	CAR	CD19	Autologous T-cells	Lentiviral vector	MAY13	APR29	Leukemia<br>Lymphoma	54
City of Hope Medical Center	USA	NCT02159495	I	CAR	CD123	Autologous T-cells	Lentiviral vector	OCT14	OCT17	Adult AML	24
Baylor College of Medicine	USA	NCT01853631	I	CAR	CD19	Autologous T-cells	Retroviral vector	FEB10	FEB17	Non-Hodgkin Lymphoma<br>CLL	14
Baylor College of Medicine	USA	NCT00889954	I	CAR	HER2	Autologous EBV T-cells	Retroviral vector	MAY09	JUL15	HER2 Positive Malignancies	18
NCI	USA	NCT01593696	I	CAR	CD19	Autologous T-cells	Retroviral vector	APR12	JAN20	ALL<br>B Cell Lymphoma<br>Leukemia<br>Large Cell Lymphoma<br>Non-Hodgkin Lymphoma	48

Supplementary table 1, *de Witte et al*

Sponsor	Country	ID	Phase	Type	Target	Method	Engineering	Start date	Completion date	Condition	Enrollment
Memorial Sloan-Kettering Cancer Center	USA	NCT01860937	I	CAR	CD19	Autologous T-cells	Unknown	MAY13	MAY16	Relapsed B-Cell ALL	24
Children's Mercy Hospital Kansas City	USA	NCT01460901	I	CAR	GD2	Allogeneic T-cells	Retroviral vector	OCT11	OCT13	Neuroblastoma	3
Children's Hospital of Philadelphia	USA	NCT01626495	I	CAR	CD19	Autologous T-cells	Lentiviral vector	AUG11	AUG16	B Cell Leukemia<br>B Cell Lymphoma	20
NCI	USA	NCT02215967	I	CAR	BCMA	Autologous T-cells	Unknown	AUG14	APR18	Myeloma, Plasma-Cell<br>Myeloma-Multiple	38
City of Hope Medical Center	USA	NCT02208362	I	CAR	CD19	Autologous T-cells	Lentiviral vector	DEC14	DEC18	Glioma	44
Memorial Sloan-Kettering Cancer Center	USA	NCT01044069	I	CAR	CD19	Autologous T-cells	Unknown	JAN10	JAN15	Leukemia<br>ALL	40
Memorial Sloan-Kettering Cancer Center	USA	NCT00466531	I/II	CAR	CD19	Autologous T-cells	Lentiviral & retroviral vector	MAR07	DEC15	Leukemia	30
Baylor College of Medicine	USA	NCT01192464	I	CAR	CD30	Autologous EBV+ T-cells	Retroviral vector	MAR11	OCT18	Hodgkin's Lymphoma<br>Non-Hodgkin's Lymphoma	18
M.D. Anderson Cancer Center	USA	NCT02194374	I	CAR	ROR1	Autologous T-cells	Unknown	DEC14	DEC8	Leukemia	48
Baylor College of Medicine	USA	NCT00586391	I	CAR	CD19	Autologous T-cells	Retroviral vector	FEB09	FEB18	B Cell Lymphoma<br>Chronic Lymphocytic Leukemia	54
City of Hope Medical Center	USA	NCT02051257	I	CAR	CD19	Autologous T-cells	Lentiviral vector	MAY14	JUN21	Leukemia<br>Lymphoma	24
City of Hope Medical Center	USA	NCT02146924	I	CAR	CD19	Autologous T-cells	Lentiviral vector	JUL14	JUL18	B-cell Adult Acute Lymphoblastic Leukemia<br>Recurrent Adult Acute Lymphoblastic Leukemia	24
City of Hope Medical Center	USA	NCT02153580	I	CAR	CD19	Autologous T-cells	Lentiviral vector	SEP14	SEP17	Leukemia<br>Lymphoma	48

Supplementary table 1, *de Witte et al*

Sponsor	Country	ID	Phase	Type	Target	Method	Engineering	Start date	Completion date	Condition	Enrollment
Abramson Cancer Center of the University of Pennsylvania	USA	NCT01837602	I	CAR	cMet	Autologous T-cells	Unknown	APR13	APR17	Metastatic Breast Cancer<br>Triple Negative Breast Cancer	15
National Cancer Institute (NCI)	USA	NCT01218867	I/II	CAR	VEGFR2	Autologous CD8+ T-cells	Retroviral vector	OCT10	OCT18	Metastatic Cancer<br>Metastatic Melanoma<br>Renal Cancer<br>Colorectal Cancer<br>Ovarian Cancer<br>Lung Cancer	118
Baylor College of Medicine	USA	NCT00840853	I/II	CAR	CD19	CMV, adenovirus, and EBV+ Autologous T-cells	Retroviral vector	APR09	APR16	ALL, CLL, Non-Hodgkin's Lymphoma	36
National Cancer Institute (NCI)	USA	NCT01583686	I/II	CAR	mesothelin	Autologous T-cells	Retroviral vector	MAR12	MAR19	Metastatic Cancer<br>Pancreatic Cancer<br>Mesothelioma<br>Ovarian	136
Abramson Cancer Center of the University of Pennsylvania	USA	NCT01897415	I	CAR	mesothelin	Autologous T-cells	Unknown	JUL13	JAN15	Metastatic Pancreatic Ductal Adenocarcinoma	10
Memorial Sloan-Kettering Cancer Center	USA	NCT01140373	I	CAR	PSMA	Autologous T-cells	Unknown	JUN10	JUN15	Prostate Cancer	18
University College, London	EU	NCT01621724	I/II	TCR	WT1	Autologous T-cells	Retroviral vector	APR12	APR16	AML, CML	18
Uppsala University	EU	NCT02132624	I/II	CAR	CD19	Autologous T-cells	Retroviral vector	APR14	APR17	B Cell Lymphoma<br>B Cell Leukemia	15
University of Zurich	EU	NCT01722149	I	CAR	FAP	Autologous CD8+ T-cells	Retroviral vector	APR13	OCT14	Malignant Pleural Mesothelioma	6
King's College London	EU	NCT01818323	I	CAR	ErbB1	Autologous CD4+ T-cells	Unknown	JUN13	JUN15	Head and Neck Cancer	30

Supplementary table 1, *de Witte et al*

Sponsor	Country	ID	Phase	Type	Target	Method	Engineering	Start date	Completion date	Condition	Enrollment
Professor Robert Hawkins	EU	NCT01493453	I	CIR (CAR)	CD19	Autologous T-cells	Retroviral vector	MAR08	MAY14	Non-Hodgkin Lymphoma	24
University College, London	EU	NCT01195480	I/II	CAR	CD19	Autologous T-cells	Retroviral vector	MAY12	DEC14	ALL	30
